# A remarkable new species of the rove beetle genus *Anthobium* Leach, 1819 from Eocene Baltic amber (Coleoptera, Staphylinidae, Omaliinae)

**DOI:** 10.3897/zookeys.973.53940

**Published:** 2020-10-05

**Authors:** Alexey V. Shavrin, Shûhei Yamamoto

**Affiliations:** 1 Institute of Life Sciences and Technologies, Daugavpils University, Vienibas 13, Daugavpils, LV-5401, Latvia Daugavpils University Daugavpils Latvia; 2 The Hokkaido University Museum, Kita 8, Nishi 5, Kita-ku, Sapporo 060-0808, Japan Hokkaido University Museum Kita Japan

**Keywords:** *
Anthobium
*, Anthophagini, fossil, Omaliini, palaeontology, sexual dimorphism, x-ray micro-CT

## Abstract

An unusual new omaliine species, *Anthobium alekseevi***sp. nov.**, is described and illustrated from Eocene Baltic amber, tentatively placed in the megadiverse genus *Anthobium* Leach, 1819. A new monotypic species-group is established. The new species can easily be distinguished from other species of the genus by the larger body, shape of the subrectangular pronotum, and the presence of a median carina on the prosternum and large, subtriangular tooth on the inner side of each mesotibia, likely exhibiting a peculiar sexual dimorphism in the male. Based on the study of the specimen with support of microtomographic images, a brief comparative analysis of a new species with described extant species of *Anthobium* is provided.

## Introduction

The rove beetles of the subfamily Omaliinae, with about 1700 species in 118 extant and 14 extinct genera, are distributed in northern temperate areas, with greatest diversity in the Holarctic region. Omaliines are common in various types of biotopes. An overwhelming number of species are hygrophiles, and they can be found near swamps, banks of rivers, mountain streams at high elevations, etc. Most species are predators of small invertebrates or are sapro- and mycophagous, and some species are even pollen-feeders (e.g. *Amphichroum* Kraatz, 1858).

The fossil history of Omaliinae was briefly discussed by Chatzimanolis (2018) and in more detail by Shavrin and Yamamoto (2019). Several extinct taxa were described based on impressions from the Jurassic (Tikhomirova 1968; Ryvkin 1985; Cai and Huang 2013), Cretaceous (Ryvkin 1990), or Cenozoic eras (Scudder 1900). Recently, seven species in three tribes of Omaliinae were reported from Eocene Baltic amber (Zanetti et al. 2016; Shavrin and Yamamoto 2019), but only one of them, *Geodromicus balticus* Shavrin & Yamamoto, 2019, belongs to the tribe Anthophagini.

Due to the poor visibility of several main details of the body, the single specimen of a possible omaliine, as an inclusion within a piece of the Baltic amber from the collection of V. Alekseev (Kaliningrad), was difficult to attribute to any taxon, and therefore it was not included in our last study on fossil omaliines (Shavrin and Yamamoto 2019). However, we recently obtained microtomographic images of this specimen, which allowed us not only to ascertain that it belongs to the subfamily Omaliinae but even to assign to the tribe Anthophagini. Based on the combination of morphological characters such as the general shape of the convex body, shapes of two preapical palpomeres of maxillary palps, the presence of postocular ridge, the shape of gular structures, located close together, the structure of the pronotum with deep mediolateral pits, and explanate lateral portions, this species clearly belongs to the *Anthobium* group of genera (e.g. Campbell 1987; Shavrin and Smetana 2017). This group contains 11 extant genera distributed in the Holarctic region: *Acidota* Stephens, 1829, *Anthobioides* Campbell, 1987, *Anthobiomorphus* Shavrin & Smetana, 2020, *Anthobium* Leach, 1819, *Arpedium* Erichson, 1839, *Camioleum* Lewis, 1893, *Caucanthobium* Assing, 2018, *Deinopteroloma* Jansson, 1946, *Deliphrosoma* Reitter, 1909, *Deliphrum* Erichson, 1839, and *Olophrum* Erichson, 1839. In addition, this group contains two extinct genera, which were described on impressions: Mesozoic *Mesodeliphrum* Ryvkin, 1990 from Turga, Transbaikal Russia (Ryvkin 1990) and *Sinanthobium* Cai & Huang, 2013 from the Middle Jurassic Jiulongshan Formation at Daohugou, Inner Mongolia, China (Cai and Huang 2013). Based on the shape of the body, features of sculpture of the forebody, the presence of long grooves in front of the ocelli (tentorial pits), the proportions of palpomeres of the maxillary palp, and the structure of the setation of the meso- and metatibia, the new species can be tentatively attributed to the megadiverse genus *Anthobium*. The senior author is currently actively exploring this genus and has published already several papers on a few established groups of species from Eastern Palaearctic Region (Shavrin and Smetana 2017, 2018, 2019; Shavrin 2020). To date, 70 species (plus four *nomina dubia*) of the genus are known from the Holarctic Region: 19 species from the western and 38 from the eastern Palaearctic regions, and 13 species from the Nearctic Region. The revision of the entire genus is still in progress and the extant taxonomic diversity has not yet been fully revealed, as several groups and species are still awaiting to be described and its phylogeny thoroughly investigated. Meanwhile, despite some morphological differences from other known taxa, the new species is nevertheless assigned to *Anthobium*, as a separate group of species. The new species represents the second species of Anthophagini known from Eocene Baltic amber. The obtained palaeontological data will undoubtedly be useful in constructing possible phylogenetic relationships in the *Anthobium* group of genera in the future.

## Materials and methods

The studied material is housed in the private collection of Vitalii I. Alekseev (Kaliningrad, Russia) and eventually will be deposited in Borissiak Paleontological Institute of the Russian Academy of Sciences, Moscow, Russia (PIN). This piece of amber was collected by net in the Baltic Sea (Kaliningrad Area, Russia) in the beginning of 2017. It was polished, embedded in a block of GTS-polyester resin, and polished again according the method of Hoffeins (2001).

All measurements are given in millimeters and were made with a stereoscopic microscope equipped with an ocular micrometer. Measurements were made from the dorsal side of the specimen except for ocular length and width of the abdomen which were made from the ventral side. Measurement of the total length of the body was difficult to do because of the specimen’s orientation within the amber piece; the resulting approximate values are marked with “~”. The type labels are cited in inverted commas and separated from each other by a comma, different lines in labels are separated with ‘|’; explanations of the type labels are given in square brackets, necessary notes within the label are given in angle brackets.

The specimen was examined using a Nikon SMZ 745T stereomicroscope. A Sony Alpha DSLR-A300digital camera was used for photographs of amber, habitus, and its details. Micro-CT observations of the specimens were conducted at the Daugavpils University (Daugavpils, Latvia) using Zeiss Xradia 510 Versa system. Scans were performed with a polychromatic x-ray beam at an energy of 40 kV and power of 3 W. Sample-detector distance was set to 17.6 mm and source to sample distance 32.6 mm. Tomographic slices were generated from 3001 rotation steps through a 360-degree rotation, using a 4× objective, and exposure time during each projection was set to 3 s. Acquired images were binned (2 × 2 × 2) giving a voxel size of 4.3 μm. Images were imported into Dragonfly PRO (ver. 4.1) software platform for interactive segmentation and 3D visualization. Prior to the full scan a 23-minute warmup scan was conducted with the same scan parameters except rotation steps which had been reduced to 201 and exposure time which was reduced to 1 s.

## Systematic palaeontology

### Order Coleoptera Linnaeus, 1758

#### Family Staphylinidae Latreille, 1802

**Subfamily Omaliinae MacLeay, 1825**

**Tribe Anthophagini Thomson, 1859**

##### Genus Anthobium

Taxon classificationAnimaliaColeopteraStaphylinidae

Leach, 1819

DD78521F-179B-5626-A1F5-AADDF6A4DB6B

###### Type species.

*Omalium atrocephalum* Gyllenhal (= *Silpha melanocephalum* sensu Marsham), for details see Herman (2001).

##### Anthobium alekseevi

Taxon classificationAnimaliaColeopteraStaphylinidae

group of species

99CDB8C5-C608-52ED-80C5-F2B90D190FC4

###### Diagnosis.

Body medium-sized; forebody convex, shiny; apical segment of maxillary palp twice as long as preceding segment; anterior angles of subrectangular pronotum slightly protruded anteriad, mediobasal third of pronotum with oval impression; lateral edges of pronotum without crenulation; prosternum with distinct median carina; surface of elytra without elevations; inner side of each mesotibia with large tooth in middle.

###### Species included.

†*Anthobium alekseevi* sp. nov.

###### Remarks.

*Anthobium alekseevi* sp. nov. differs from the remaining species of the genus by the larger body, the shape of the subrectangular pronotum, the presence of distinct median carina on the prosternum, and the presence of a large median tooth on the inner side of the mesotibia.

##### † Anthobium alekseevi

Taxon classificationAnimaliaColeopteraStaphylinidae

Shavrin & Yamamoto sp. nov.

F63B5670-9B34-5E01-9B4B-7FD3C750BD7A

http://zoobank.org/A1797770-BE2C-4B9A-99B1-5D93D54B7943

Figures 1–4 , 5, 6 , 7, 8 , 9–13 , 14–19

###### Type material examined.

**Figures 1–4.**
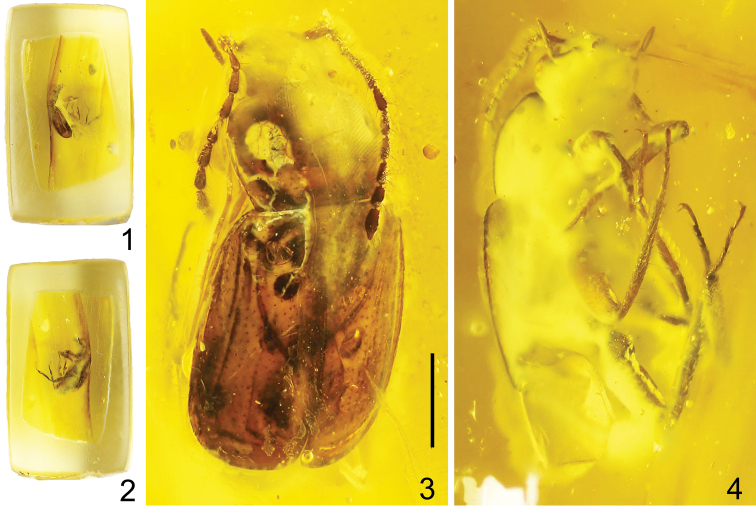
*Anthobium alekseevi* sp. nov. **1, 2** amber specimen with inclusion **3** habitus, dorsal view **4** habitus, dorsoventral view. Scale bar: 1.0 mm (**3, 4**).

**Holotype**: male, complete specimen as inclusion in a piece of small yellow Baltic amber, 11.0 mm × 0.7 mm × 0.5 mm in size (Figs 1, 2), with glued small paper on side of an amber labeled “AWI148” and additional labels within a plastic envelope: “AWI-148 | Omaliinae | gen. nov. | (mesotibia!)” <handwritten>, “**HOLOTYPE** | *Anthobium* | *alekseevi* sp. nov. | Shavrin A. & Yamamoto S. des. 2020” <red rectangular label, printed> (PIN).

###### Preservation.

The specimen is poorly visible because it is partially covered with white microbubbles, and some details of the structure of the body are not visible: head, median portion of pronotum and scutellum, ventral side of the body and abdomen (Figs 1, 2). The basal part of the abdomen ventrally is covered by left hind wing.

###### Locality and horizon.

Baltic amber from Kaliningrad Area, westernmost Russia; mid-Eocene (ca 44 Ma; Wappler 2005).

###### Description.

Measurements: maximum width of head including eyes: 1.30; length of head (from base of labrum to neck constriction along head midline in dorsal view): 0.75; ocular length: 0.40; length × width of segments III and IV of maxillary palpi: III: 0.15 × 0.10, IV: 0.30 × 0.10; length of antenna: 2.70; length of pronotum: 1.35; maximum width of pronotum: 1.75; sutural length of elytra from apex of scutellum to posterior margin of sutural angle: 2.60; length of elytron from basal to apical margin: 2.95; maximum width of elytra: 2.30; length of metatibia: 1.60; length of metatarsus: 0.80; maximum width of abdomen (at segment IV): 2.10; total length (from anterior margin of clypeus to apex of abdomen): ~5.40.

Body oblong, moderately wide, shiny (Fig. 3); body laterally as in Figures 5, 6, 9, and 10; body dorsolaterally as in Figure 4; body ventrally as in Figures 11 and 12; forebody as in Figure 14. Body and antennomeres 3–11, brown; legs and mouthparts reddish-brown; antennomeres 1 and 2 yellow-brown.

**Figures 5, 6.**
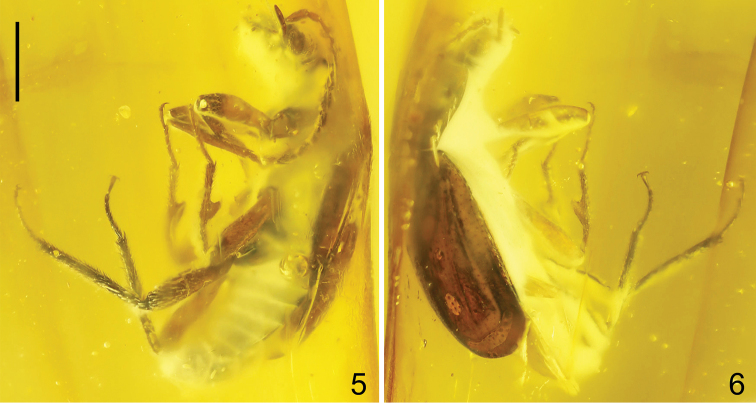
*Anthobium alekseevi* sp. nov., habitus (lateral view). Scale bar: 1.0 mm.

Head transverse (Figs 15, 16), 1.7 times as wide as long; anterior portion of frons with obliquely elevated supra-antennal prominences and moderately wide impressions behind them; middle portion slightly elevated, with distinct, deep, narrow dorsal tentorial pits (grooves) in front of ocelli, diagonally stretching apicad to level of middle length of eyes; basal portion with distinct, narrow impression between ocelli; postocular ridge distinct, acute, located relatively close to posterior margin of eye, if see laterally (Figs 5, 9, 18). Eyes large, convex. Ocelli large, situated at about level of postocular ridges; distance between ocelli slightly shorter than distance between ocellus and posterior margin of eye (Figs 15, 16). Labrum wide, transverse. Mentum and labium narrow, with distinctly elongate apical labial palpomere (Fig. 12). Preapical segment of maxillary palpus moderately long, 1.5 times as long as wide, and as wide as apical segment; apical segment about twice as long as preceding segment, from middle gradually narrowing toward apex (Figs 5, 11, 18). Gular sutures with shortest distance located at level of posterior third of eyes (Fig. 12). Antenna moderately short, exceeding apical third of elytra, with elongate antennomeres 5–10 and long preapical setae, antennomeres 3–11 covered by dense pubescence; basal antennomere moderately wide, 1.5 times as long as 2, antennomere 2 ovoid, about twice as long as wide and slightly shorter than 3, 3 and 4 as wide as 2, 5–7 slightly shorter than 4, 8 and 9 slightly shorter than 7, 10 distinctly shorter than 9, apical antennomere 1.6 times as long as 10, from middle gradually narrowed toward apex (Fig. 3).

Pronotum subrectangular, 1.2 times as wide as long, 1.3 times as wide as head, widest in middle, evenly rounded both anteriad and posteriad (Fig. 14); apical margin rounded, distinctly narrower than posterior margin; anterior angles widely rounded, slightly protruded anteriad; posterior angles obtuse; lateral edges bordered, without visible crenulation; disc with middle portion widely elevated, with indistinct, wide, semioval impression on mediobasal third, and lateral portions moderately wide, slightly explanate, each with deep pit in middle (Fig. 16). Laterobasal and basal portions of pronotum with dense and fine punctation (Fig. 3). Pronotal hypomeron and postcoxal process well developed; intercoxal process elongate and moderately wide; prosternum with distinct median carina (Figs 11, 18). Scutellum large and wide, with rounded apex. Metaventrite wide, convex.

Elytra convex, slightly longer than wide, about twice as long as pronotum, indistinctly widened in middle, reaching basal margin of abdominal tergite VI, with widely rounded apical margins (Fig. 17); shoulders of elytra rounded; lateral portions narrow, explanate; surface of elytra without visible elevations. Punctation sparse and moderately small, each elytron with longitudinal rows of vague serial punctures in middle (Fig. 3). Hind wings fully developed.

**Figures 7, 8.**
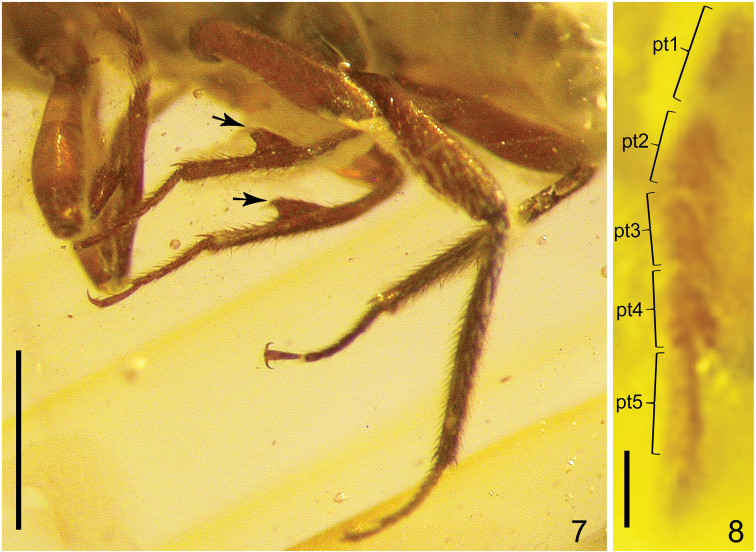
*Anthobium alekseevi* sp. nov. **7** legs **8** protarsus. Scale bars: 1.0 mm (**7**); 0.1 mm (**8**). Abbreviations: pt1–pt5 = protarsomeres 1–5.

Legs long (Fig. 7); procoxae wide, protruding ventrad, contiguous; mesocoxae large, convex; metacoxae strongly transverse; pro-, meso-, and metatrochanter relatively narrow, elongate (Figs 11, 12); all femora widest at about middle, profemora slightly wider than meso- and metafemora; pro- and mesotibiae about as long as femora; protibiae covered with dense long setae; protarsomeres 1–5 as in Fig. 8; mesotibiae indistinctly curved in middle, inner side of each mesotibia with large and wide subtriangular, fin-shaped tooth in middle (Fig. 7, *arrows*); meso- and metatibiae covered by very dense, long and strong setae (including apex of tooth on each mesotibia); metatibiae with additional very long setae; metatibiae distinctly longer than metafemora, slightly widened in about middle, covered by dense, strong and long setae, with a few additional spines around apical margin; all tarsi 5-segmented, all tarsi combined shorter than tibia; tarsomeres 1–4 with long and dense lateral setation; apical tarsomere about as long as preceding three tarsomeres together; tarsal claws simple, without modifications (Fig. 7).

Abdomen distinctly narrower than elytra (Fig. 17), from segment IV significantly narrowing apicad (Fig. 11); abdominal segment IX elongate. Abdomen ventrally as in Figures 11 and 12.

Male. Protarsomeres 1–4 distinctly wide, with very long lateral setae (Fig. 8). Apical margin of abdominal tergite VIII rounded (Fig. 17). Apical margin of sternite VIII sinuate (Fig. 12).

Female unknown.

###### Etymology.

Patronymic, the species is named to honor our colleague Vitalii I. Alekseev (Kaliningrad), great contributor to the knowledge of the fossil beetle fauna from Baltic amber.

**Figures 9–13.**
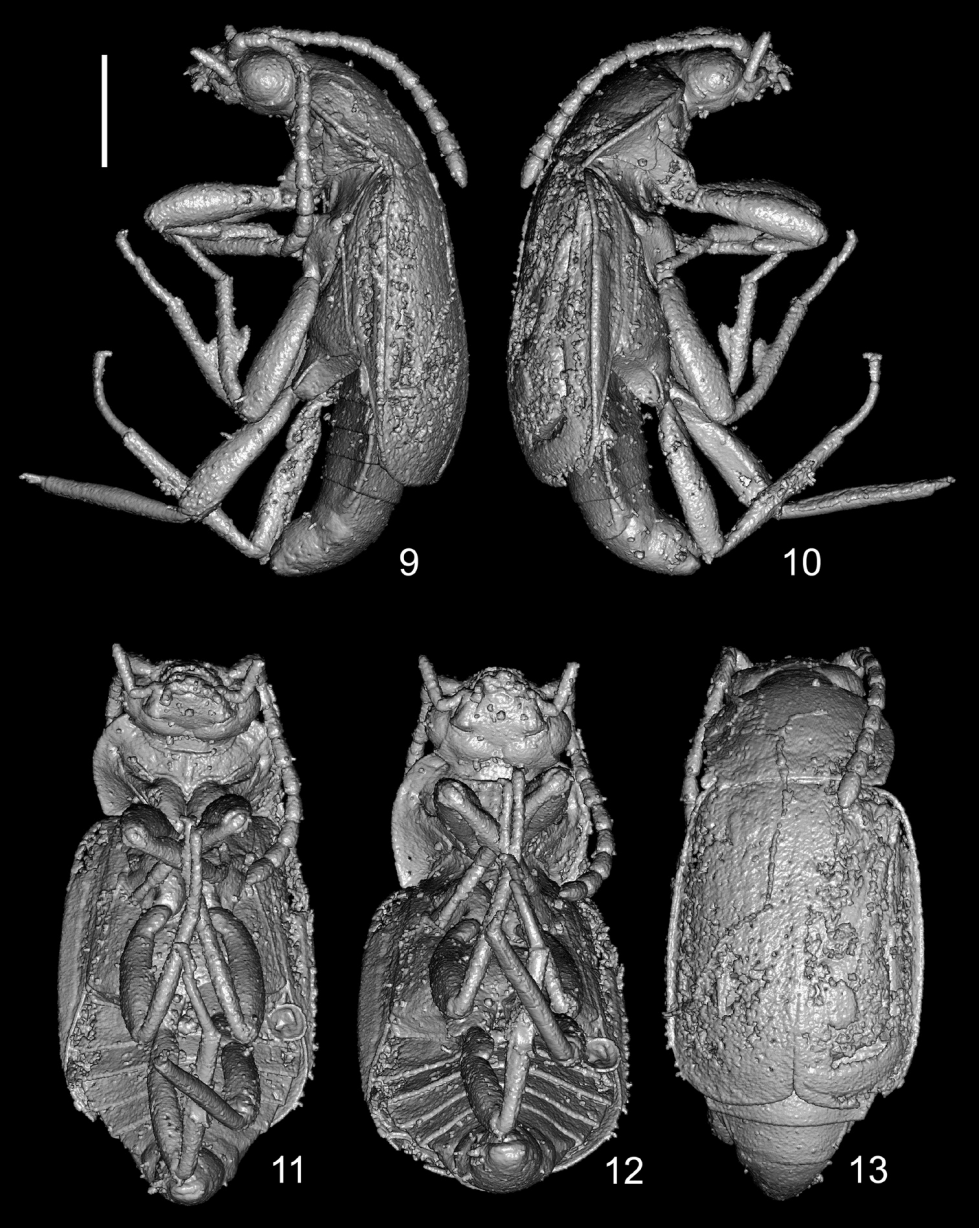
Habitus of *Anthobium alekseevi* sp. nov. **9, 10** lateral view **11, 12** ventral view **13** dorsal view. Scale bar: 1.0 mm.

## Discussion

In general, *A. alekseevi* sp. nov. can be characterized by the similar coloration and shape of the convex, shiny, and glabrous body, as in many species of *Anthobium*. The punctation of the forebody is poorly visible except in the lateral and basal portions of the pronotum and elytra; the median portion of the elytra bears longitudinal rows of punctures. The pronotum is shiny, and the microsculpture between punctures is missing. Similar punctation on the shiny forebody is specific for some East Asiatic species groups (e.g. *gracilipalpe* and *nigrum*). Similar to many species of the genus, the head of *A. alekseevi* sp. nov. has distinct median elevation and elongate grooves in front of the ocelli. Unfortunately, each latero-apical portion of the head is hidden by adjoining basal anttennomeres, so the presence and shape of an antocular identation are invisible. Usually, the antocular identation of *Anthobium* is variable in its shape: some species have distinct and semicircular notch as figured by Zanetti (1987: fig. 73a), and many species from Asia have a smooth or very indistinct notch. The shapes of the two preapical palpomeres of the maxillary palpus of *A. alekseevi* sp. nov. are similar to that of many Asiatic congeners. These palpomeres are approximately equal in their width, a characteristic shared in common with almost all the other species of the genus. The shape of the antennomeres of *A. alekseevi* sp. nov. is as usual for *Anthobium*, with the elongate antennomeres 5–10 recalling some species from Asia (e.g. *Anthobium daliense* Shavrin & Smetana, 2017, species of the *morchella* group). The presence of a distinct mediobasal impression on the pronotum is similar to that of some Eastern Palaearctic species. On the contrary, the smooth margins of the elytra without distinct crenulation is suggestive of species from the Western Palaearctic Region. The microtomography of the specimen has not shown the presence of the aedeagus within the abdomen. Usually, species of *Anthobium* have the median lobe variable in width, narrow and long parameres, and a simple internal sac, sometimes with sclerotized additional structures.

*Anthobium alekseevi* sp. nov. has several significant morphological features that distinguish it from other known species of the genus, which led us to propose a new, separate species group for it. The first peculiar feature is the large body of the new species, which is roughly 5.4 mm long. So far, the largest specimens of *Anthobium* have been 4.75 mm long: Himalayan *A. nigrum* (Cameron, 1924) and Chinese *A. puetzi* Shavrin & Smetana, 2017. On average, the body length of known species of the genus varies from 3.0 to 4.0 mm, and the smallest species is the Chinese *A. liliputense* Shavrin & Smetana, 2018, with specimens as small as 1.8 mm in body length. The second peculiar feature is the shape of subrectangular pronotum (Fig. 17). The pronotum of known species of *Anthobium* is distinctly transverse, and the minimum width of it in some species is usually 1.4 times as wide as long. The third peculiar one is the presence of a distinct median carina on the prosternum (Figs 11, 18). Most species of *Anthobium* have no similar structure on the prosternum. A similar structure can be found in some species of *Arpedium*, which have an indistinct carina-like elevation in the middle of the prosternum (see Campbell 1984: figs 14, 15). And finally, one of the interesting features of the new species is the presence of highly modified mesotibia, which possess a very large tooth in the middle (Fig. 7). Various modifications of the male tibia are frequent in some Anthophagini. A small subtriangular tooth or large triangular dilatation on inner margin of the protibia are known in some species of *Anthobium* (e.g. *A. gracilipalpe* (Champion, 1920); *A. unicolor* (Marsham, 1802), and *A. atrocephalum* (Gyllenhal, 1827); see Palm 1948: figs 155f, 156f), *Arpedium* (see Campbell 1984: figs 60, 64), *Camioleum* (see Shin and Ahn 2006: fig. 1) or *Olophrum* (e.g. *Olophrum tadashii* Watanabe, 1990; see Watanabe 1990: fig. 134). Some species have different modifications of the metatibia, such as the very long and acute, median, spine-shaped protrusion on inner side of each metatibia of *Trichodromeus armatus* (Cameron, 1941) (see Coiffait 1983: fig. 2M). Some species of *Amphichroum* are characterized by a deep and wide indentation in the middle of metatibia, sometimes with an indistinct median tooth (see Zanetti 1987: fig. 86). The inner sides of the pro- and mesotibia of *Anthobioides* have a large, convex swelling on their apical portion (see Campbell 1987: figs 1, 34, 35). In light of the examples above, *A. alekseevi* sp. nov. has one of the most unusual sexual modifications of the mesotibia within the tribe.

**Figures 14–19.**
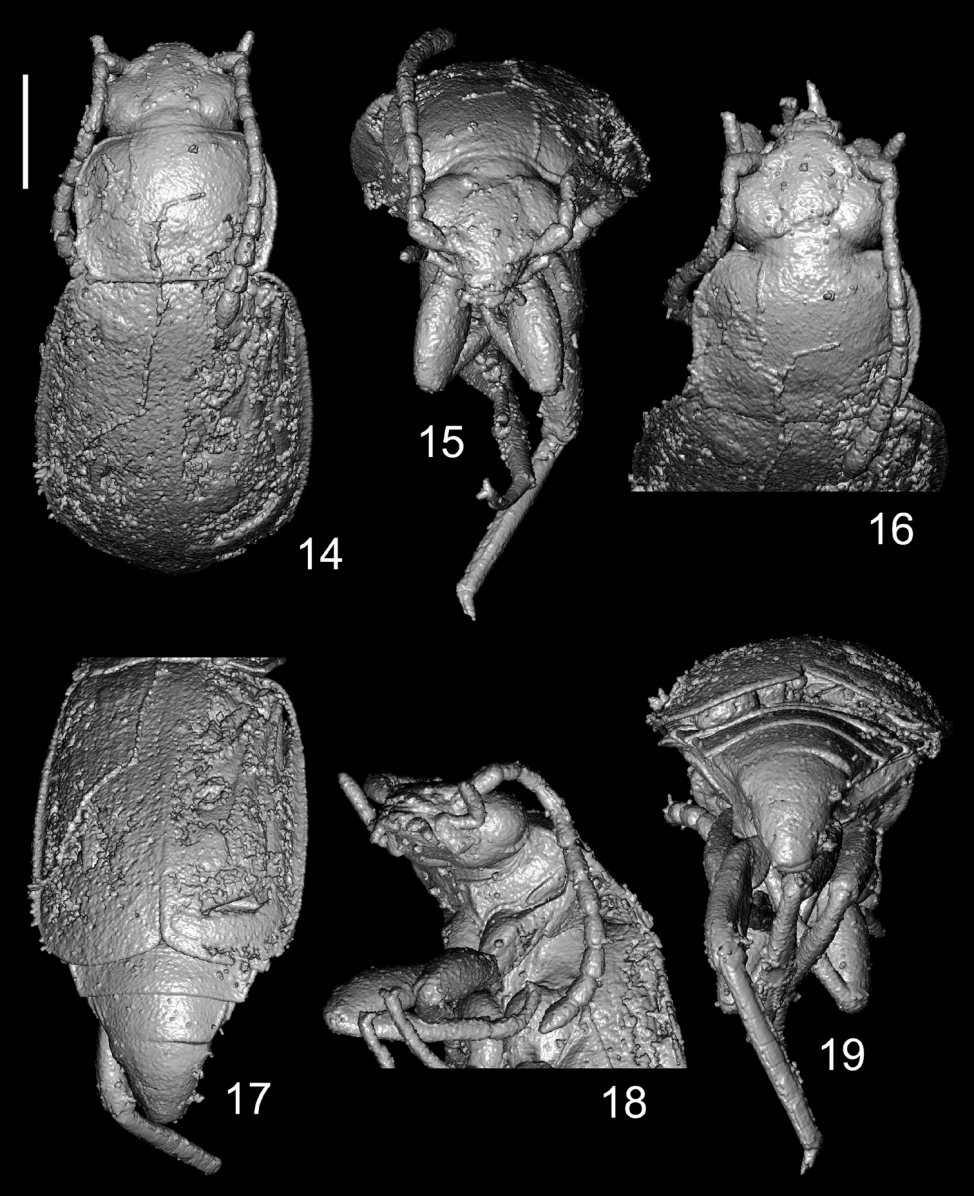
*Anthobium alekseevi* sp. nov. **14** forebody, dorsal view **15** head, apical portion of pronotum and legs, frontal view **16** head, pronotum and antenna, dorsal view **17** elytra and abdomen, dorsal view **18** head and thorax, lateroventral view **19** apical portion of elytra, abdomen and legs, posterodorsal view. Scale bar: 1.0 mm.

Species of *Anthobium* are strongly dependent on a temperate climate, living in forest litter and wet moss, and most commonly inhabit wet habitats near swamps and along banks of streams and rivers. Hypothetically, *A. alekseevi* sp. nov. may have lived in wet biotopes near rivers or swamps. A similar temperate-loving, extinct, and potentially rheophilous species, *Geodromicus balticus* Shavrin & Yamamoto, 2019, was also described from Eocene Baltic amber.

## Supplementary Material

XML Treatment for Anthobium

XML Treatment for Anthobium alekseevi

XML Treatment for Anthobium alekseevi
